# Utilizing ultra-early continuous physiologic data to develop automated measures of clinical severity in a traumatic brain injury population

**DOI:** 10.1038/s41598-024-57538-5

**Published:** 2024-03-31

**Authors:** Shiming Yang, Peter Hu, Konstantinos Kalpakis, Bradford Burdette, Hegang Chen, Gunjan Parikh, Ryan Felix, Jamie Podell, Neeraj Badjatia

**Affiliations:** 1 Program in Trauma, University of Maryland School of Medicine, 22. S. Greene Street, G7K19, Baltimore, MD 21201 USA; 2 Department of Anesthesiology, University of Maryland School of Medicine, Baltimore, USA; 3 Department of Computer Science and Electrical Engineering, University of Maryland, Baltimore County, Baltimore, USA; 4 Department of Epidemiology and Public Health, University of Maryland School of Medicine, Baltimore, USA; 5 Department of Neurology, University of Maryland School of Medicine, Baltimore, USA; 6 Fischell Department of Bioengineering, University of Maryland, College Park, USA

**Keywords:** Traumatic brain injury, Glasgow coma scale, Injury severity score, Mortality, Machine learning, Brain injuries, Machine learning

## Abstract

Determination of prognosis in the triage process after traumatic brain injury (TBI) is difficult to achieve. Current severity measures like the Trauma and injury severity score (TRISS) and revised trauma score (RTS) rely on additional information from the Glasgow Coma Scale (GCS) and the Injury Severity Score (ISS) which may be inaccurate or delayed, limiting their usefulness in the rapid triage setting. We hypothesized that machine learning based estimations of GCS and ISS obtained through modeling of continuous vital sign features could be used to rapidly derive an automated RTS and TRISS. We derived variables from electrocardiograms (ECG), photoplethysmography (PPG), and blood pressure using continuous data obtained in the first 15 min of admission to build machine learning models of GCS and ISS (ML-GCS and ML-ISS). We compared the TRISS and RTS using ML-ISS and ML-GCS and its value using the actual ISS and GCS in predicting in-hospital mortality. Models were tested in TBI with systemic injury (head abbreviated injury scale (AIS) ≥ 1), and isolated TBI (head AIS ≥ 1 and other AIS ≤ 1). The area under the receiver operating characteristic curve (AUROC) was used to evaluate model performance. A total of 21,077 cases (2009–2015) were in the training set. 6057 cases from 2016 to 2017 were used for testing, with 472 (7.8%) severe TBI (GCS 3–8), 223 (3.7%) moderate TBI (GCS 9–12), and 5913 (88.5%) mild TBI (GCS 13–15). In the TBI with systemic injury group, ML-TRISS had similar AUROC (0.963) to TRISS (0.965) in predicting mortality. ML-RTS had AUROC (0.823) and RTS had AUROC 0.928. In the isolated TBI group, ML-TRISS had AUROC 0.977, and TRISS had AUROC 0.983. ML-RTS had AUROC 0.790 and RTS had AUROC 0.957. Estimation of ISS and GCS from machine learning based modeling of vital sign features can be utilized to provide accurate assessments of the RTS and TRISS in a population of TBI patients. Automation of these scores could be utilized to enhance triage and resource allocation during the ultra-early phase of resuscitation.

## Introduction

A central challenge in the early management of traumatic brain injury (TBI) is the lack of accurate and timely systems for triage and prognostication^1^. Though the Glasgow coma scale (GCS) is widely considered a robust measure of injury severity in isolated TBI (isoTBI), there are significant limitations to its accuracy^2^. The pupillary light reflex (PLR) is a well-validated biomarker in pre-hospital settings; however, the PLR can be confounded by factors such as age^3^, concomitant facial trauma or administration of pharmacological agents^4^. Additional injury specificity provided by CT imaging may be delayed or limited in resource challenged environments.

By contrast, continuous vital sign monitoring with electrocardiogram (ECG), photoplethysmography (PPG), and blood pressure monitoring is universally applied and can provide a wealth of useful information. Time and frequency domain features of PPG and ECG-based heart rate variability (HRV) have been identified as early indicators of secondary injury in TBI^5,6^. These features are highly sensitive markers of autonomic nervous system dysfunction and are understandably dynamic during the early resuscitation phase after injury. We believe a composite of these features can approximate the clinical phenotype expressed as the GCS in TBI patients and can provide an estimation of the constellation of injuries that account for the injury severity score (ISS). The GCS and ISS are integral features used for calculating the revised trauma score (RTS) and trauma injury severity score (TRISS), both of which have been shown to be accurate assessments of in-hospital mortality after TBI^7^. In this study we sought to analyze the first 15 min of continuous vital sign data obtained from the time of admission to the hospital to develop an automated calculation of the RTS and TRISS based on machine-learning derived assessments of the GCS and ISS.

## Methods

### Study population

This is a single-center, retrospective study conducted at the R Adams Cowley Shock Trauma Center (STC) at the University of Maryland Medical Center. This study was approved by the Institutional Review Board (IRB) of the University of Maryland School of Medicine.

**Figure 1:**
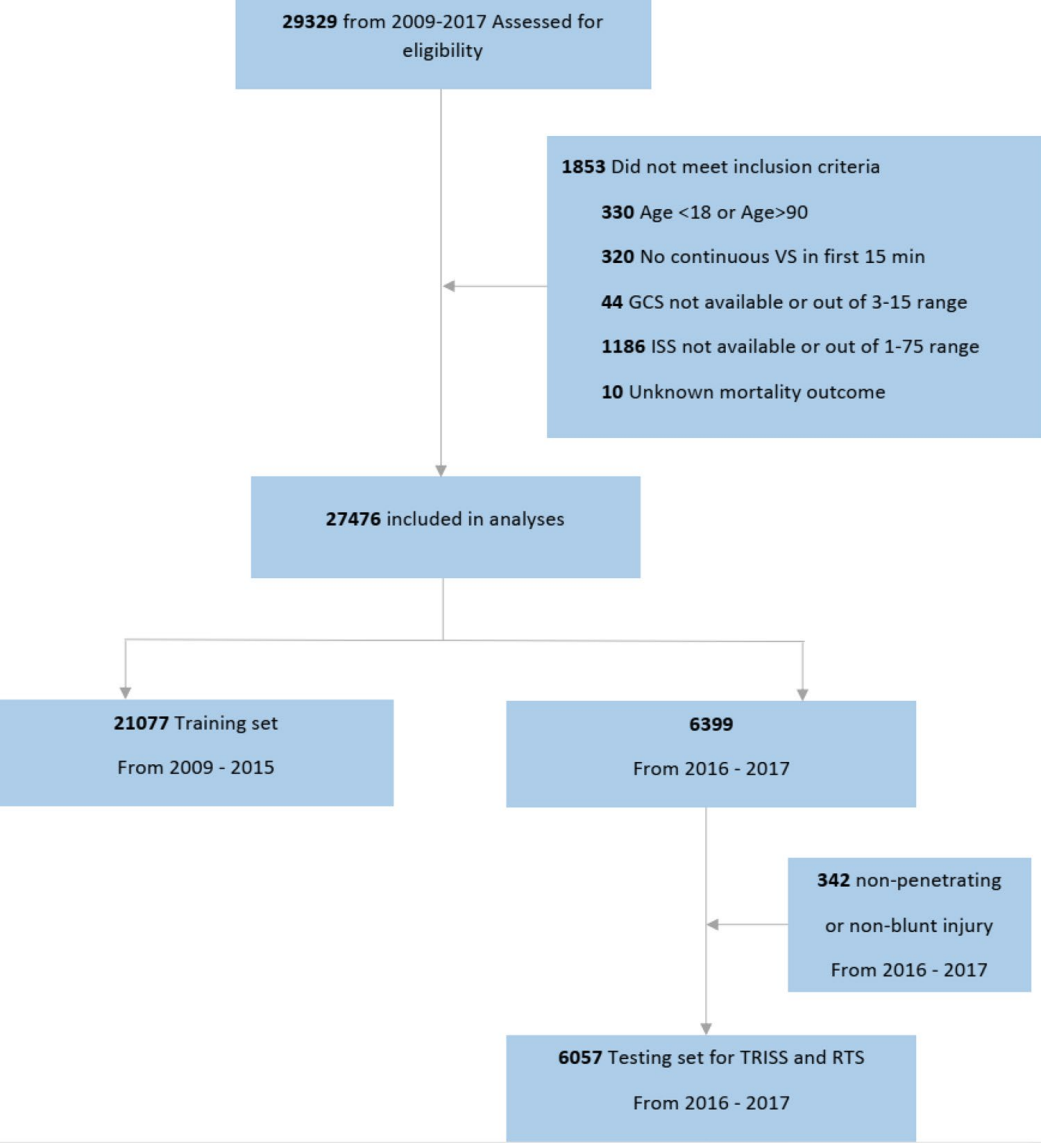
Flow Diagram for patient selection.

**Figure 2:**
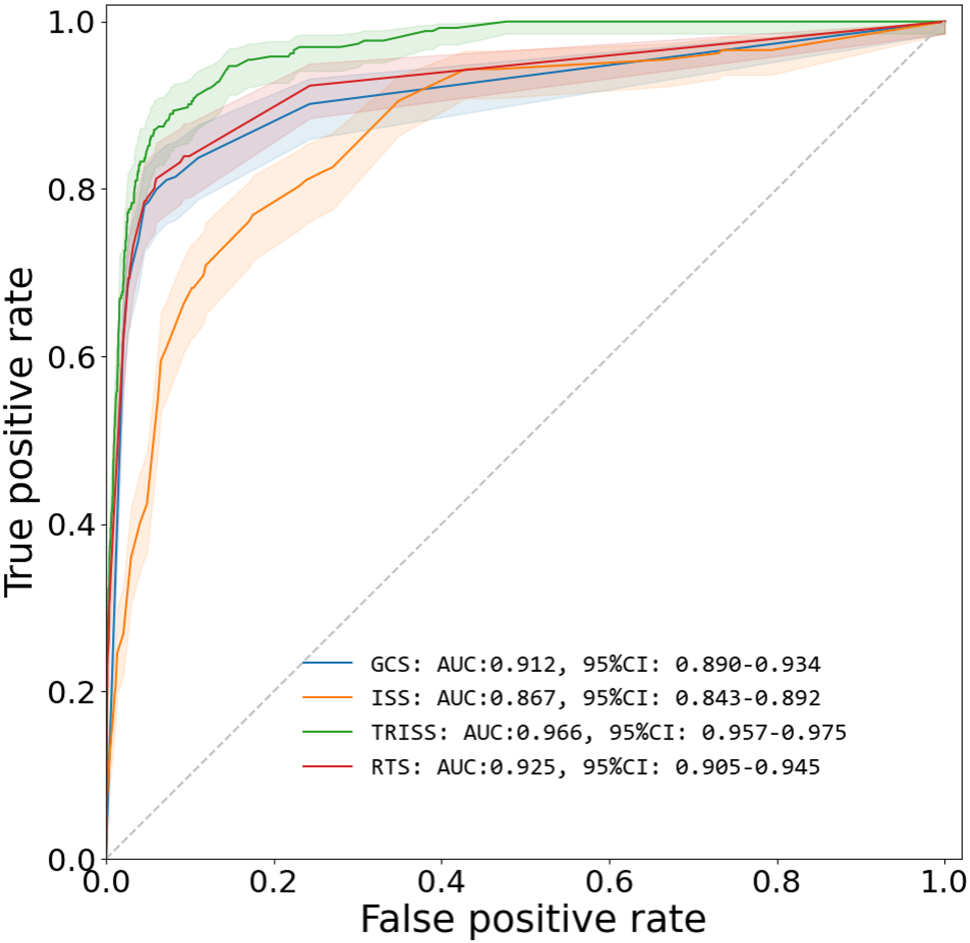
Mortality prediction using GCS, ISS, TRISS, RTS.

This study screened adult trauma patients (age between 18 and 90 years old) directly admitted to STC from the scene of injury between 2009 and 2017. Baseline demographic and clinical data was obtained from the institutional trauma registry. Injury severity score (ISS) was calculated on every patient after the primary survey and information from initial imaging studies were compiled. In our hospital trauma registry, patients discharged between 2009 and 2013 were coded in AIS-2005, while those discharged after 2013 were coded in AIS-1990. Patients were excluded if they died within 15 min after TRU admission, if there were less than 5 min of continuous VS recorded, if their GCS/ISS values were unavailable, or if their mortality outcome was missing. For the dataset used for testing, patients were excluded if they did not sustain either blunt or penetrating injuries because the TRISS model is calculated only for those two types of injuries. Figure 1 shows the flow diagram for patient selection. Within the general trauma population, we specifically focused on TBI with systemic trauma (polyTBI: head abbreviated injury scale (AIS) ≥ 1) and isolated TBI (isoTBI: head AIS ≥ 1 and other AIS ≤ 1) subgroups (Fig. 2).

### Physiologic data collection and processing

Continuous vital sign (VS) data were collected in the 13-bay Trauma Resuscitation Unit (TRU) from the networked patient monitors (GE-Marquette-Solar-7000/8000, GE Healthcare, Fairfield, CT). Each monitor collects 240 Hz waveforms (e.g., ECG and PPG) and 0.5 Hz numeric data, including heart rate (HR), oxygen saturation (SpO2), blood pressures (BP), and among others^8^.

During resuscitation, outliers and noise are added to data due to patient movement or loose attachment of sensors. We filtered our extreme values that are outside of the sensors’ measurement ranges or reasonable physiologic ranges (HR > 200 bpm, SBP > 250 mmHg, DBP > 200 mmHg). We used a median filter with 30 s window length to smooth numeric VS. For waveforms, we applied a robust smoothing algorithm to reduce noise, which is based on a penalized least squares method and allows fast smoothing of data by means of a discrete cosine transform^9^.

### Model development

We built separate models for estimating GCS and ISS using the variables derived from the first 15 min of continuous VS in TRU. Considering the cardinalities of GCS and ISS, we modeled GCS as a multi-class classification problem and ISS as a regression problem for their exact values instead of coarse categories. To achieve autonomous prediction, we primarily used variables derived from the continuous VS. VS variables included descriptive statistics of numeric VS within 15 min after TRU admission, such as mean, standard deviation (SD), median, and interquartile range (IQR)^10^. Dose (integrated area normalized by time duration) of VS above or below normal thresholds for heart rate (HR > 120 bpm or HR < 60 bpm), SpO2 < 88%, mean arterial blood pressure (MBP < 50 mmHg), and shock index (SI = SBP/HR > 1)^11^. PPG and ECG-based heart rate variability (HRV) time and frequency domain features were calculated using standard definitions based on the Task Force of the European Society of Pacing and Electrophysiology^12^. Frequency domain features were calculated using three distinct popular methods, including Welch’s method of averaging periodograms from overlapping intervals, analysis of least-squares based Lomb periodograms, and parametric autoregressive modeling^13–15^. Non-linear dynamics HRV features included measures of entropy, Poincare plots, and fractal analyses^16^.

The modeling outcomes are on TRU admission GCS and ISS. These are discrete integer values, with imbalanced classes for each value in our dataset. Mild TBI (GCS 13–15) was about 30 times more frequent than moderate TBI (GCS 9–12) and 15 times more frequent than severe TBI (GCS 3–8). Similarly, mild ISS (1–8) was about twice as frequent as moderate ISS (9–15) and severe ISS and above (> 15). To reduce the effects of class imbalance, we inversely weighted each case proportional to its class’s frequency. When calculating their frequencies, we coarsened GCS into mild, moderate, and severe TBI categories. For ISS, we used mild, moderate, severe, and profound (ISS > 24) categories (Table 1).

**Table 1:** Clinical characteristics.

Characteristics	Training (N = 21,077)	Testing		
		All (N = 6057)	Poly-TBI (N = 3278)	Isolated TBI (N = 1760)
Age mean (SD)	42.7 (18.7)	43.4 (19.0)	45.1 (19.4)	45.4 (19.5)
Female	6420 (30.5%)	1696 (28.0%)	950 (29.0%)	561 (31.9%)
Mechanism of Injury				
Blunt	17,169 (81.5%)	3561 (58.8%)	2004 (61.1%)	1108 (63.0%)
Penetrating	3173 (15.1%)	2496 (41.2%)	1274 (38.9%)	652 (37.0%)
Admission GCS (Median[IQR])	15 [15]	15 [14, 15]	15 [14, 15]	15 [14, 15]
Mild (13–15)	19,013 (90.2%)	5362 (88.5%)	2734 (83.4%)	1543 (87.7%)
Moderate (9–12)	726 (3.4%)	223 (3.7%)	162 (4.9%)	90 (5.1%)
Severe (3–8)	1338 (6.4%)	472 (7.8%)	382 (11.7%)	127 (7.2%)
ISS (Median [IQR])	5 [5, 14]	5 [2, 14]	9 [5, 17]	5 [2, 5]
Mild (1–8)	11,394 (54.1%)	3322 (54.8%)	1626 (49.6%)	1456 (82.7%)
Moderate (9–15)	4743 (22.5%)	1517 (25.0%)	808 (24.6%)	180 (10.2%)
Severe (16–24)	2644 (12.5%)	682 (11.3%)	458 (14.0%)	46 (2.6%)
Profound (> 24)	2296 (10.9%)	536 (8.9%)	386 (11.8%)	78 (4.5%)
Mortality	716 (3.4%)	264 (4.4%)	186 (5.7%)	58 (3.3%)
Time to death (hours, Median[IQR])	17.1 [1.9, 105.3]	10.6 [2.7, 80.3]	27.0 [3.2, 91.7]	30.1 [5.8, 79.6]

For the machine learning algorithms, we compared the Extreme Gradient Boosting Tree (XGBoost), Random Forest (RF), and linear regression (LR with ElasticNet penalty). XGBoost is an efficient implementation of boosting tree, for both GCS classification and ISS regression. In the following, we refer to the two models ML-GCS and ML-ISS^17^. Boosting tree is a machine learning method that often achieves superior prediction performance on tabular data. It can account for complex non-linearity and high-order interactions. It is robust to outliers in and multicollinearity among variables. It can perform variable selection through tree pruning and regularization. We split the dataset into two major parts. The cases from 2009 to 2015 were used for model training, and the cases from 2016–2017 were used for testing the models’ performance on unseen new data. During training, we tuned XGBoost model hyperparameters including the total number of trees, maximum tree depth, learning rate, and percentage of sampled variables. For the ElasticNet model, we tuned the hyperparameters the weight of penalty terms, and the ratio between L1 and L2 penalties^18^. Optimal hyperparameters were identified via randomized search using five-fold cross-validation with 10 replicates.

### Evaluation

First, we evaluated the ML-GCS and ML-ISS against the ground truth in the testing dataset for polyTBI and isoTBI subgroups. For ML-GCS, we used receiver operating characteristic (ROC) areas under the curve (AUC) for each class (3–15). We also used the macro-averaged and micro-averaged AUCs. Confusion matrices were used to show the accuracy of ML-GCS and ML-ISS in different categories. For model interpretation, we analyzed the variables’ contribution to the models’ prediction to gain insights. The SHapley Additive exPlanations (SHAP) values were used to calculate variable importance by comparing model predictions with and without the variable^19^. The variable importance at a global level is given by adding the absolute value of the SHAP values for each individual data point. SHAP values were calculated and displayed graphically in order to describe each variable’s contribution to the outcome prediction and improve model interpretability. Positive SHAP values indicate that a variable increases the model output relative to its expected value, while negative SHAP values indicate that a variable decreases the model output relative to its expected value.

Next, we plugged the estimated GCS and ISS into TRISS and RTS scoring systems as surrogate values and evaluated the scores’ prediction performance for in-hospital mortality. Specifically, we calculated ML-RTS by using ML-GCS. We calculated ML-TRISS by using ML-ISS and real GCS. In addition, we compared a similar ML-TRISS2 by using ML-ISS and ML-GCS. ROC curves, AUROCs, and their 95% confidence intervals (CIs) were used to compare scores’ prediction performance. True positive rate (TPR), true negative rate (TNR), positive predictive value (PPV), negative predictive value (NPV), accuracy, and F-1 score were reported based on the threshold that maximized the Youden index^20^.

## Results

### Study population

A total of 29,329 patients from 2009 to 2017 were assessed for eligibility. There were 1,853 cases that did not meet the inclusion criteria. 21,077 cases from 2009 to 2015 were used for model training, including internal cross-validation for hyperparameter tuning. Among them, 30.5% were female. The mean age was 42.7 years old (SD = 18.7). The majority (90.2%) had mild TBI (GCS 13–15). The median ISS was 5 and the IQR was 5–14, with 54.1% being mild.

There were 6,399 cases from 2016–2017 that satisfied the overall inclusion criteria. Since we also evaluated the ML-GCS in the TRISS score, which is defined for patients with blunt or penetrating injuries, we further removed cases that were not blunt or penetrating in the testing dataset. A total of 6,057 cases were used for testing. Among them, 28.0% were female. The mean age was 43.4 years old (SD = 19.0). There were 88.5% mild TBI category and 54.8% mild ISS. The testing set had a higher percentage of penetrating injury (41.2%) than those in the training set (15.1%). In the testing dataset, there were 3,278 polyTBI and 1,760 isoTBI cases. The polyTBI group had a higher proportion of moderate (4.9%) and severe (11.7%) TBI those in the training set (3.4% and 6.3%). It also had a higher mortality rate (5.7%) than those in the training set (3.4%).

### Evaluation with ground truth GCS/ISS

**Figure 3:**
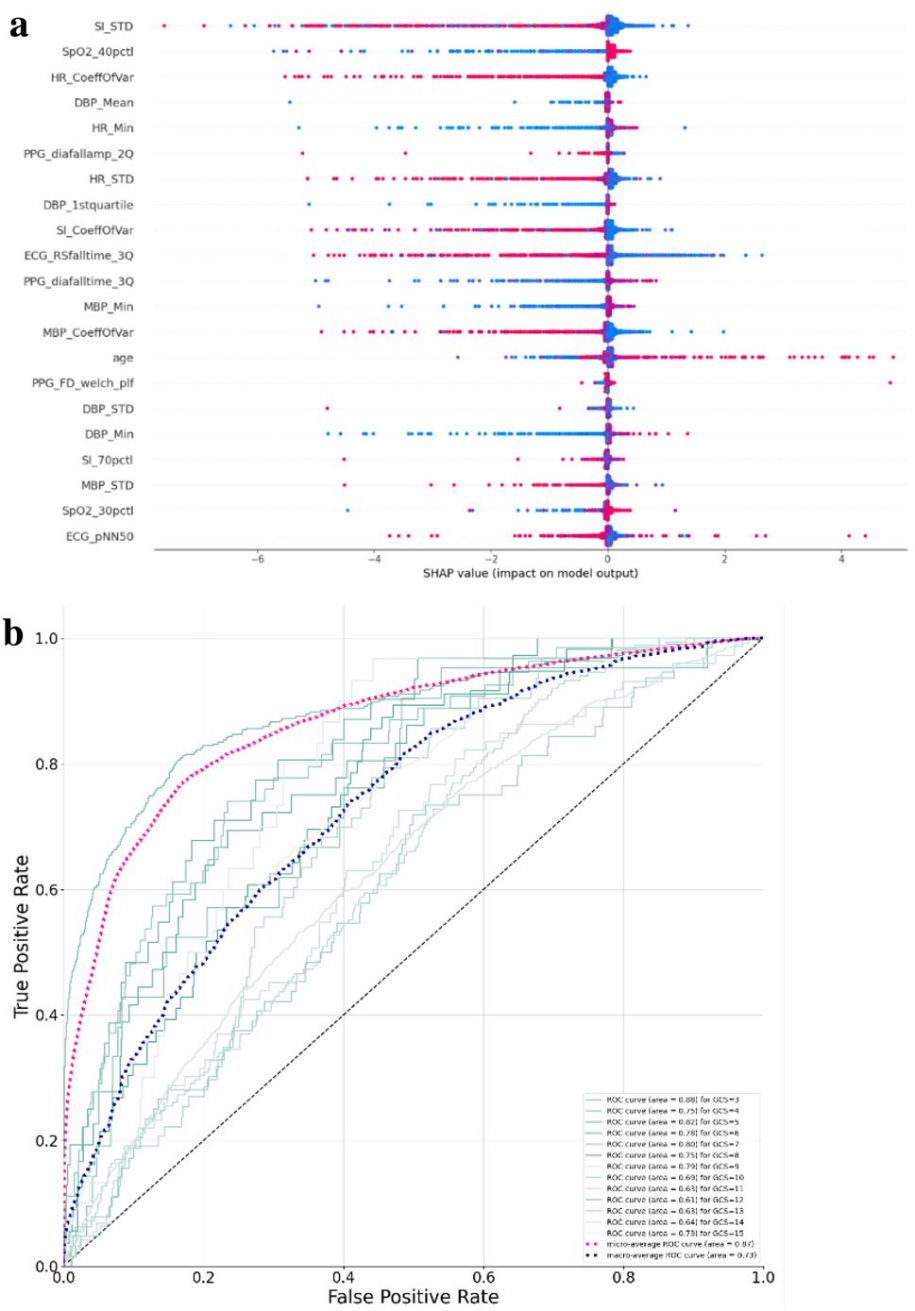
(**a**) SHAP output for GCS. (**b**) ROC for multi-class GCS prediction.

With the trained models for estimating GCS and ISS, we evaluated their performance in the testing datasets against the ground truth. Figure 3b shows ROC curves for the ML-GCS model in estimating classes for the testing dataset (N = 6057). The curves were generated by comparing each class to all the others. A macro-average will compute the metric independently for each class and then take the average (hence treating all classes equally), whereas a micro-average will aggregate the contributions of all classes to compute the average metric. In a multi-class classification setup, micro-average is preferable for class imbalance. For ML-GCS, it performed relatively better in distinguishing GCS = 3 or not (AUC = 0.88). It performed poorly in discriminating GCS = 12 or not (AUC = 0.61). The micro-average AUC is 0.87. Using the confusion matrix, we aggregated GCS into five categories, which followed the GCS points definition in RTS (Figure S2). The ML-GCS had a TPR of 58.16% for category 0 (GCS = 3). It also had a high TPR of 71.15% for category 4 (GCS = 13–15).

**Figure 4:**
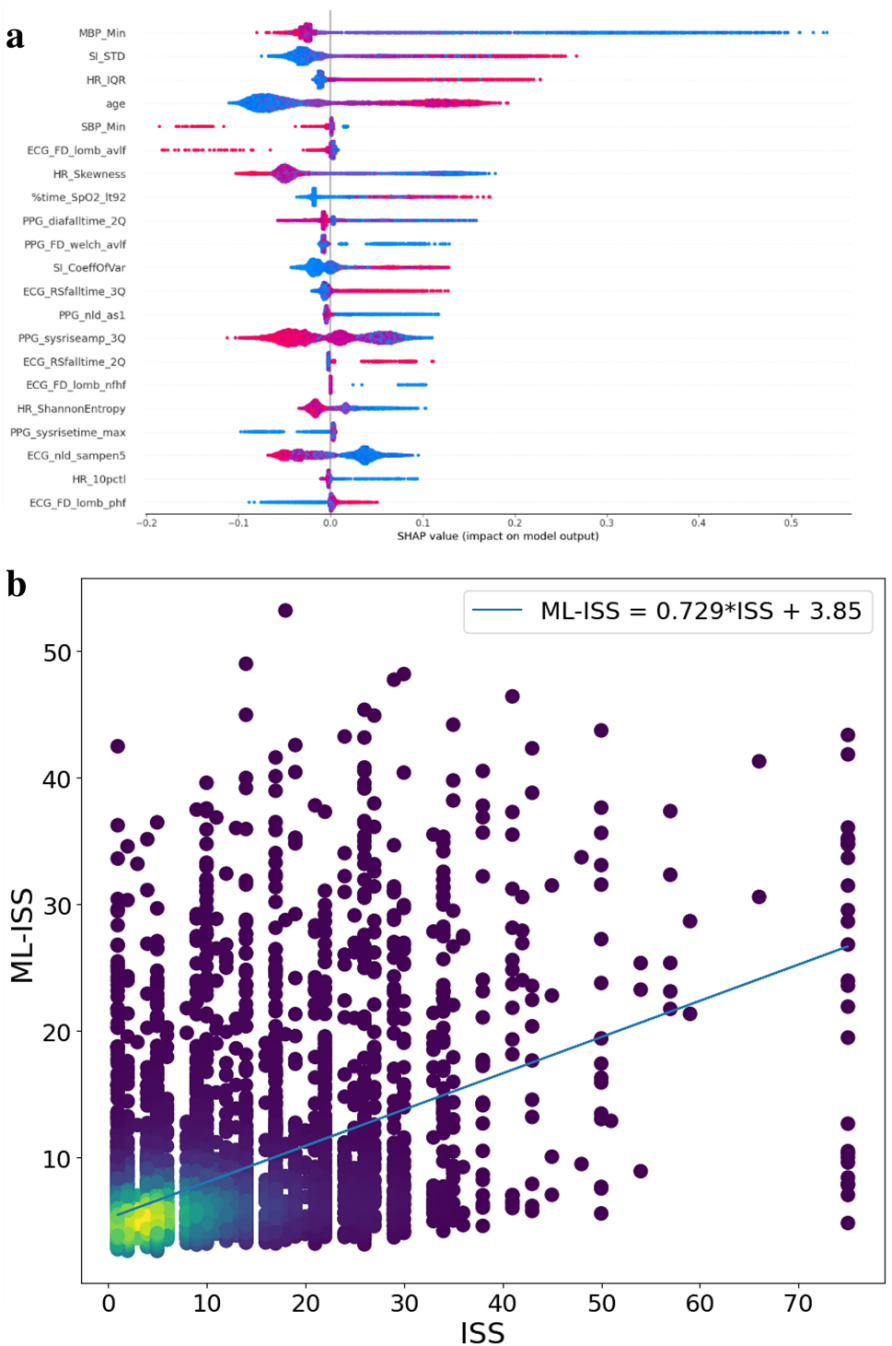
(**a**) SHAP output for ISS. (**b**) Regression plot for ISS prediction.

For linear regression ML-ISS, the model tends to underestimate real ISS (Fig. 4b). We aggregated ISS into four categories, mild, moderate, severe, and profound. The ML-ISS had a TPR of 86.24% for the mild. However, it tended to underestimate other categories as mild. For example, 73.37% of moderate cases were estimated as mild (Figure S2).

### Evaluation with mortality prediction

**Figure 5:**
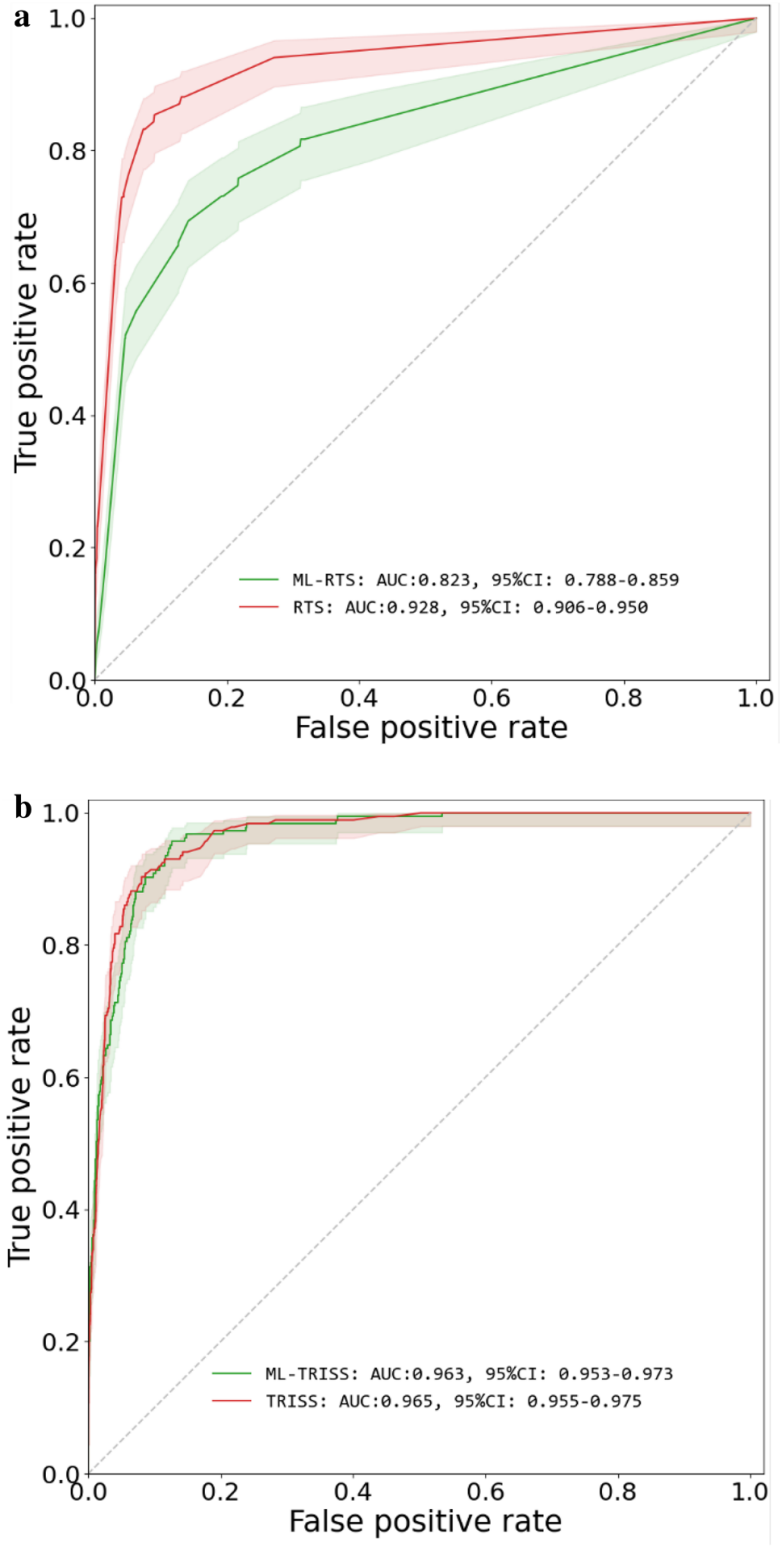
(**a**) Mortality prediction for RTS vs ML-RTS in Poly-TBI, (**b**) Mortality Prediction for TRISS vs ML-TRISS in Poly-TBI.

**Figure 6:**
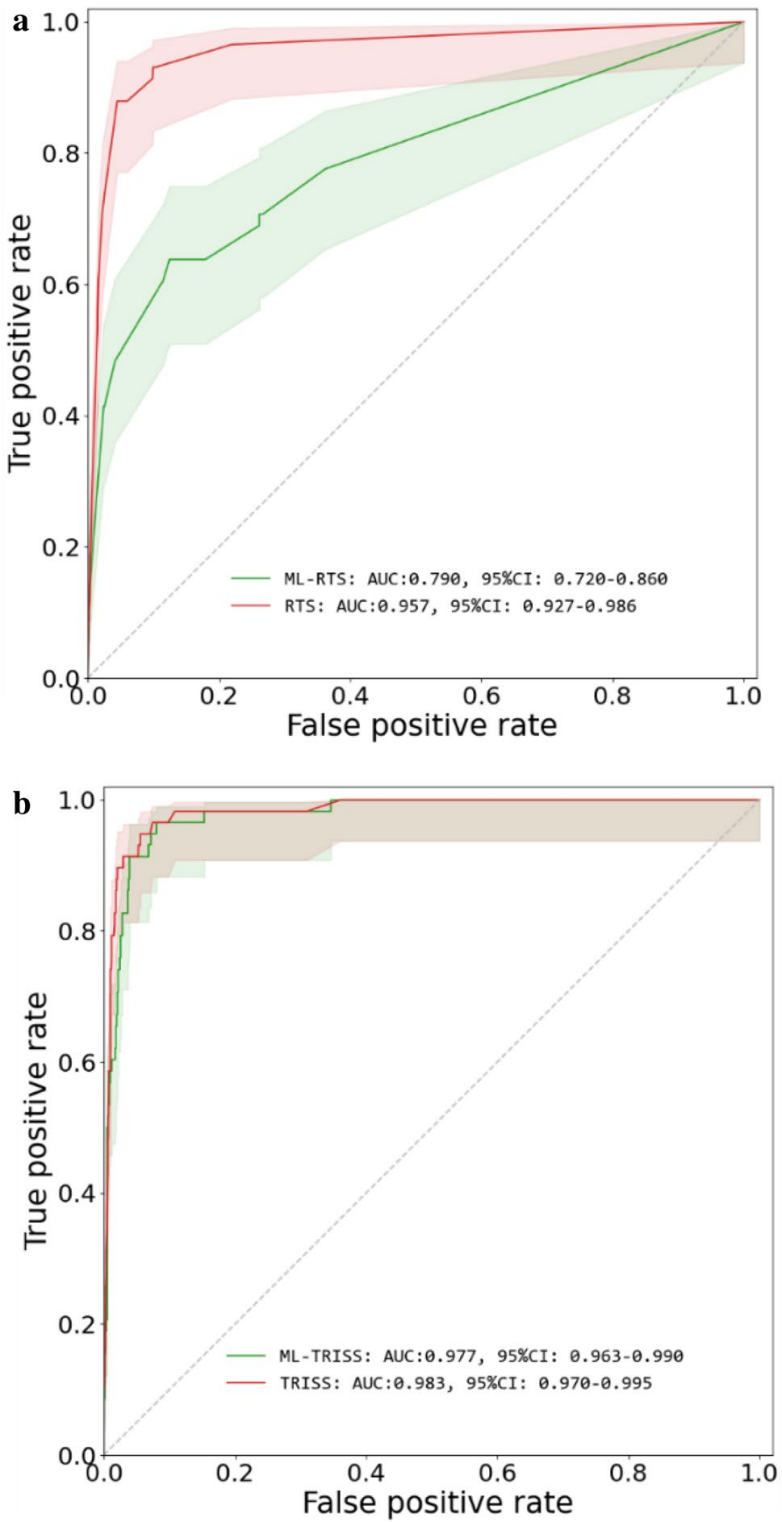
(**a**) Mortality prediction for RTS vs ML-RTS in isolated TBI. (**b**) Mortality Prediction for TRISS vs ML-TRISS in isolated TBI.

We evaluated ML-GCS and ML-ISS in scoring systems that use GCS and ISS as components. For the polyTBI group, the ML-RTS had AUC 0.823 (95%CI 0.788–0.859). The real RTS had AUC 0.928 (95%CI 0.906–0.950). ML-TRISS had AUC 0.963 (95%CI 0.953–0.973), while the real TRISS had AUC 0.965 (95%CI 0.955–0.975) (Fig. 5). For the isoTBI group, the ML-RTS had AUC 0.79 (95%CI 0.72–0.86). The real RTS had AUC 0.957 (95%CI 0.927–0.986). ML-TRISS had AUC 0.977 (95%CI 0.963–0.990), while the real TRISS had AUC 0.983 (95%CI 0.970–0.995) (Fig. 6).

### Model interpretation

Variable importance analysis was performed to show the variable contributions to the prediction. Figure 3a shows the variable importance for the XGBoost model of predicting GCS = 3. Figure 3b shows the variables for the XGBoost model of ISS. Each dot represents a variable’s value for a patient. The color spectrum from red to blue stands for the variable’s value ranging from high to low. The x-axis is the SHAP value, which represents how greatly knowing that variable’s value influences the log-odds. In this plot we can see that lower minimum mean blood pressure (MBP) had the highest impact to increase the model’s prediction of ISS, followed with SI standard deviation, inter-quartiles of HR, and age (Fig. 3b).

## Discussion

In this study of over 27,000 consecutively admitted trauma patients we leveraged physiologic information obtained from continuous monitoring to develop machine learning-based estimates of GCS and ISS. In testing the ability of the ML-ISS values to replace actual ISS values, the ML-TRISS was noted to perform as well as the TRISS in the prediction of in-hospital mortality (Figs. 5b, 6b) for both isoTBI and polyTBI patients. The ML-RTS, which utilized ML-GCS in place of actual GCS did not perform as well as RTS or TRISS in the prediction of in-patient mortality, though the overall accuracy for ML-RTS and ML-TRISS2 was still relatively high (Table 3). Importantly, all these models were built on PPG, ECG, and blood pressure data obtained during the first 15 min after arrival to the hospital, prior to the availability of any additional laboratory or imaging data, illustrating the ability to develop an automated severity scoring system during the ultra-early resuscitation stages after injury.

We compared three different learning algorithms, XGBoost, random forest, and linear regression (with an ElasticNet penalty), to estimate GCS and ISS. The results showed that XGBoost and RF had similar performance and were significantly better than the linear model when predicting mortality for the polyTBI and isoTBI patients.

This work has significant clinical implications for the field or early in-hospital care of TBI patients. Advances in sensing and computational techniques make it possible for continuous vital signs to be collected and processed without human intervention. By automating the calculation of established mortality risk scores with the addition of ML-GCS and ML-ISS variables, clinicians may be able to identify patients at risk for poor outcomes during the early resuscitation phase. Rapid recognition can prompt targeted lifesaving interventions and appropriate resource allocation. This is particularly important in the remote field or in mass casualty events where clinical expertise and resources are scarce.

Previous studies have identified the utility of machine learning algorithms to enhance the ability to predict mortality early after injury. Many have combined hemodynamic measurements with laboratory data or additional clinical information gathered within hours of arrival. One recent analysis found the GCS and ISS among the features that were the most important in predicting in-hospital mortality among severe TBI patients. Our analyses differ from these reports in that we attempted to replicate only the amount of information available at the time of initial triage (within 15 min of arrival) and so intentionally did not utilize information from electronic medical records, laboratory data, or imaging findings. This allows for better interpretation in a real-world triage scenario where these data will not be immediately available or attainable.

Our modeling of ISS and GCS also highlights a method of domain adaptation as an alternative method for developing predictive algorithms^21–24^. Domain adaptation is the task of developing machine learning algorithms that can be easily transferred from one domain to another. This problem arises when there is a large collection of labeled data in one source domain but the task at hand requires developing a model that performs well in a separate target domain. The goal is to adapt the model to the target domain using as little labeled data as possible. The approach proposed in this paper is based on the idea of transforming the domain adaptation learning problem into a standard supervised learning problem^25^. The transformation is done by augmenting the feature space of both the source and target data and using the result as input to a standard learning algorithm. This transformation allows any standard algorithm to be applied to the problem, making it easy to implement and use.

Model performance was less accurate with the RTS than TRISS. The RTS score is comprised of the GCS, respiratory rate, and systolic blood pressure, with the greatest weight given to the GCS (Table 2). Because the accuracy of the ML-GCS models varied depending on the GCS score, it is not surprising that the ML-RTS score did not perform as well as the actual RTS score in predicting in-patient mortality. A similar result was noted when using the ML-GCS alongside the ML-ISS in the TRISS estimation (Supplemental Fig. 4). Regardless of these limitations, the NPV for metrics utilizing ML-GCS was no different than those metrics that utilized measured GCS (Table 3). It is also important to recognize that we chose to predict the value of ML-ISS and ML-GCS specific to in-patient mortality. Given this study included all severities of TBI at arrival with a very high preponderance of mild TBI, the mortality rate was lower than previous predictive algorithms of in-patient mortality that focused more narrowly on severe TBI.

**Table 2:** Equations and calculations for RTS, TRISS (blunt), TRISS (penetrating).

	Formulas					
RTS	0.9368*GCS points + 0.7326*SBP points + 0.2908*RR points					
TRISS blunt	1 – 1/(1 + EXP(− 0.4499 + RTS*0.8085 + ISS*-0.0835 + (IF(age > = 55,1,0)* − 1.743)))					
TRISS penetrating	1 – 1/(1 + EXP(− 2.5355 + RTS*0.9934 + ISS* − 0.0651 + (IF(age > = 55,1,0)* − 1.136)))					
Points conversion in RTS	Glasgow coma scale		Systolic blood pressure		Respiratory rate	
	GCS	Points	SBP	Points	RR	Points
	3	0	0	0	0	0
	4–5	1	1–49	1	1–5	1
	6–8	2	50–75	2	6–9	2
	9–12	3	76–89	3	> 29	3
	13–15	4	> 89	4	10–29	4

**Table 3:** Accuracy, sensitivity, specificity of ISS, GCS, TRISS, ML-TRISS, RTS, ML-RTS for in-hospital mortality.

	AUROC	AUC 95%CI	Sensitivity	Specificity	PPV	NPV	Accuracy	F1
ISS	0.867	0.843–0.892	0.769	0.825	0.167	0.987	0.822	0.274
GCS	0.912	0.890–0.934	0.799	0.940	0.377	0.990	0.934	0.513
TRISS	0.966	0.957–0.975	0.894	0.920	0.336	0.995	0.919	0.489
RTS	0.925	0.905–0.945	0.812	0.940	0.379	0.991	0.935	0.517
*ML-TRISS*	0.961	0.950–0.972	0.920	0.894	0.282	0.996	0.900	0.432
*ML-RTS*	0.855	0.828–0.883	0.722	0.880	0.215	0.986	0.873	0.331

Additional limitations to our analyses include our single center dataset, which may have been influenced by institutional practice and patient characteristics specific to our local region. Further evaluation and training with data from other institutions might be necessary to generalize the model. The GCS is a subjective assessment, based on the opinion of the clinicians and could have variability in the scores^26^. Therefore, the trained models may be subject to inaccurate labeling of the outcomes. In addition, the dataset spans 9 years, and it is possible that the data has drifted over time. Patients discharged between 2009 and 2013 were coded in AIS-2005, while those discharged after 2013 were coded in AIS-1990. The Injury Severity Score (ISS), which relies on AIS, may be calculated based on varied definitions. AIS was initially defined in 1990 and has since evolved into multiple versions, including those released in 2005 and 2015^27^. A comparison of 145 patients’ AIS-1998 and AIS-2005 revealed that AIS-2005 coded the same injuries with lower severity scores (*p* < 0.01) and decreased mean and maximum AIS-head scores (*p* < 0.01)^28^. The AIS-Head section underwent specific changes in 2005 to capture more detailed information about head injuries, such as hematoma size, to better reflect their clinical severity. The changes also aimed to improve the accuracy of coding concussive TBI and allowed for coding of hypoxic or ischemic traumatic brain injury, which was not codable in AIS-1998. These revisions may have introduced major changes in coding the severity of the same traumatic brain injuries and may have changed the types of injuries captured by AIS codes. However, the changes in AIS versions may have limited impact on TRISS calculation. Li et al. compared AIS1998 and AIS2015 in a study of 739 cases. In predicting mortality, TRISS (AIS1998) had an AUROC of 0.936, which was not significantly different from the AUROC of TRISS (AIS2015) 0.942^29^. However, given the large size of the training sample, we still expect that the model could learn useful information from the single center’s clinicians.

## Conclusion

In this large retrospective cohort study of TBI patients we demonstrate the ability to develop ML-GCS and ML-ISS estimations that can be utilized to automate the RTS and TRISS score during the ultra-early phase of resuscitation. These findings support the concept of utilizing transfer learning as a technique in machine learning (ML) to boost predictive algorithm performance.

### Supplementary Information

Supplementary Information 1.

Supplementary Information 2.

## Data Availability

The datasets used and/or analysed during the current study are available from the corresponding author on reasonable request.

## Code availability

The underlying code for this study [and training/validation datasets] is not publicly available for proprietary reasons.
