# Supplementary material for: Utilizing ultra-early continuous physiologic data to develop automated measures of clinical severity in a traumatic brain injury population
Source: Sci Rep. 2024 Mar 31;14:7618. doi: 10.1038/s41598-024-57538-5 (PMC10982286; doi:10.1038/s41598-024-57538-5)
Supplement: Supplementary file 1 — Supplementary Information 1. [file 41598_2024_57538_MOESM1_ESM.docx]

**Appendix**

**A1. ISS, GCS, TRISS, ML (XGB)-TRISS, RTS, ML(XGB)-RTS, RF-TRISS, RF-RTS, LR-TRISS, LR-RTS predictive performance for in-hospital mortality**

|  | **AUROC** | **AUC 95%CI** | **Sensitivity** | **Specificity** | **PPV** | **NPV** | **Accuracy** | **F1** |
| --- | --- | --- | --- | --- | --- | --- | --- | --- |
| **ISS** | 0.867 | 0.843-0.892 | 0.769 | 0.825 | 0.167 | 0.987 | 0.822 | 0.274 |
| **GCS** | 0.912 | 0.890-0.934 | 0.799 | 0.940 | 0.377 | 0.990 | 0.934 | 0.513 |
| **TRISS** | 0.966 | 0.957-0.975 | 0.894 | 0.920 | 0.336 | 0.995 | 0.919 | 0.489 |
| **RTS** | 0.925 | 0.905-0.945 | 0.812 | 0.940 | 0.379 | 0.991 | 0.935 | 0.517 |
| ***XGB-TRISS*** | 0.961 | 0.950-0.972 | 0.920 | 0.894 | 0.282 | 0.996 | 0.900 | 0.432 |
| ***XGB-RTS*** | 0.855 | 0.828-0.883 | 0.722 | 0.880 | 0.215 | 0.986 | 0.873 | 0.331 |
| ***RF-TRISS*** | 0.960 | 0.949-0.971 | 0.904 | 0.909 | 0.310 | 0.995 | 0.909 | 0.462 |
| ***RF-RTS*** | 0.844 | 0.816-0.872 | 0.722 | 0.860 | 0.190 | 0.986 | 0.854 | 0.301 |
| ***LR-TRISS*** | 0.932 | 0.921-0.943 | 0.897 | 0.889 | 0.267 | 0.995 | 0.889 | 0.412 |
| ***LR-RTS*** | 0.712 | 0.680-0.745 | 0.445 | 0.870 | 0.134 | 0.972 | 0.851 | 0.206 |

**A2. Comparison of AUROCs and 95%CIs of TRISS, TRISS2, RTS estimated by XGB, RF, and LR for mortality in poly-TBI and isolated TBI groups.**

|  |  | Poly-TBI | | Isolated TBI | |
| --- | --- | --- | --- | --- | --- |
| Real | TRISS | 0.965 | 0.955-0.975 | 0.983 | 0.970-0.995 |
|  | RTS | 0.928 | 0.906-0.950 | 0.957 | 0.927-0.986 |
| XGB | TRISS | 0.963 | 0.953-0.973 | 0.977 | 0.963-0.990 |
|  | TRISS2 | 0.880 | 0.853-0.907 | 0.863 | 0.808-0.917 |
|  | RTS | 0.823 | 0.788-0.859 | 0.790 | 0.720-0.860 |
| RF | TRISS | 0.961 | 0.950-0.972 | 0.934 | 0.915-0.953 |
|  | TRISS2 | 0.865 | 0.836-0.894 | 0.768 | 0.702-0.834 |
|  | RTS | 0.812 | 0.776-0.848 | 0.618 | 0.547-0.689 |
| LR | TRISS | 0.925 | 0.912-0.938 | 0.934 | 0.915-0.953 |
|  | TRISS2 | 0.774 | 0.736-0.813 | 0.768 | 0.702-0.834 |
|  | RTS | 0.626 | 0.585-0.667 | 0.618 | 0.547-0.689 |


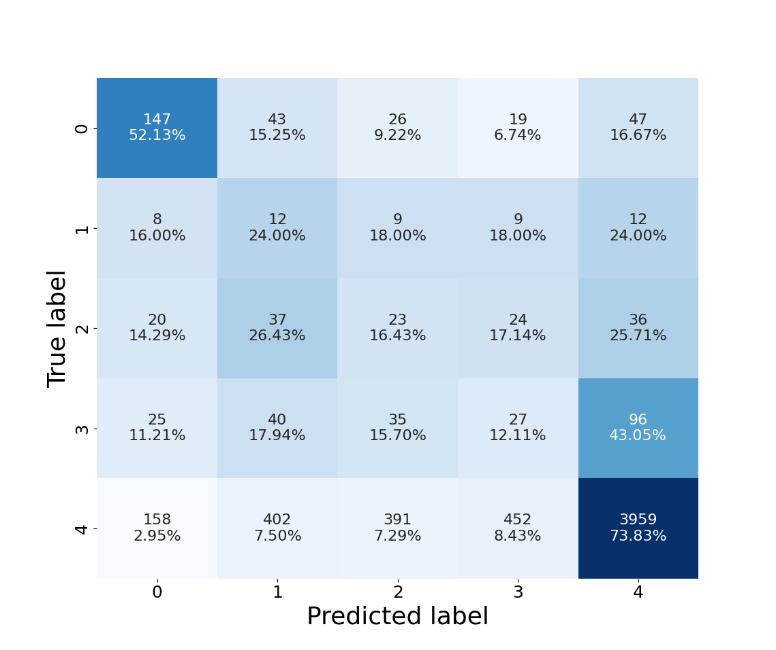

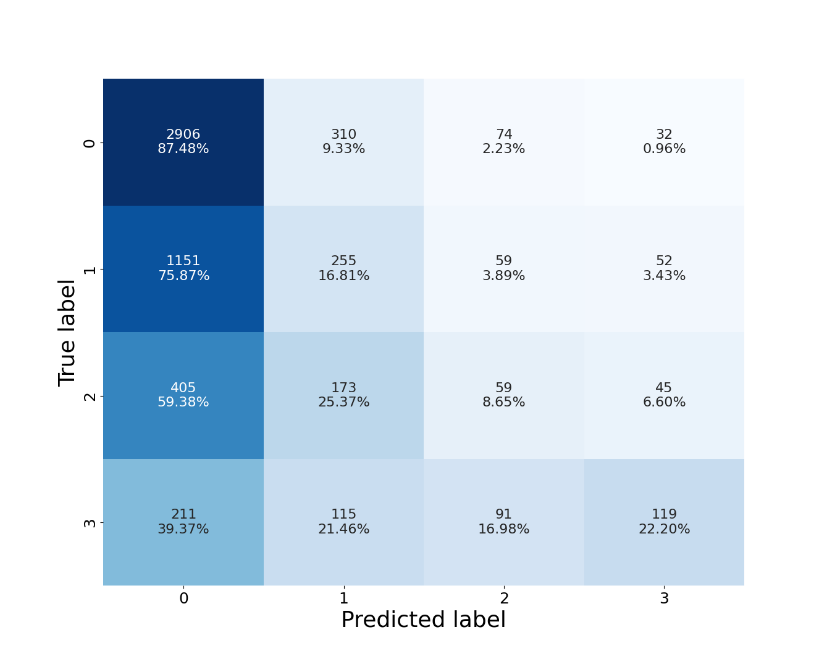


**A3. Classification matrices for GCS and ISS in all TBI patients (Random Forest)**


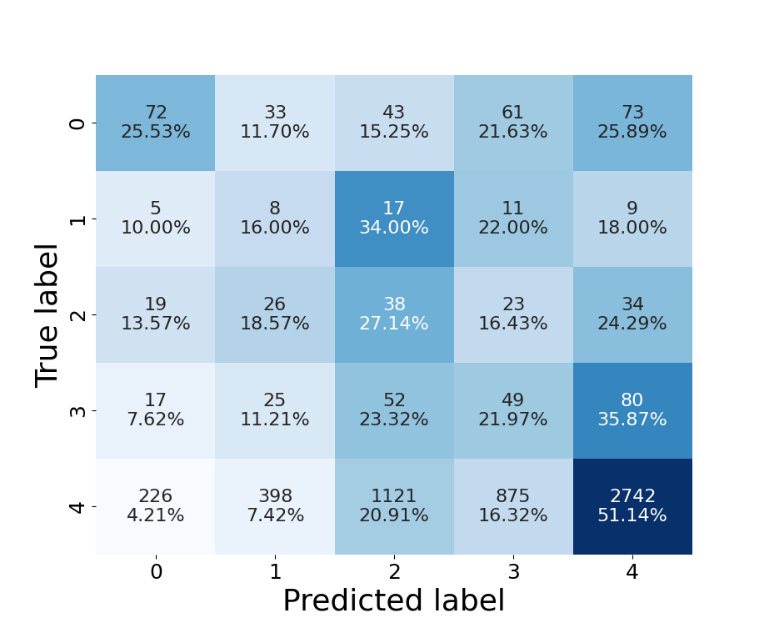

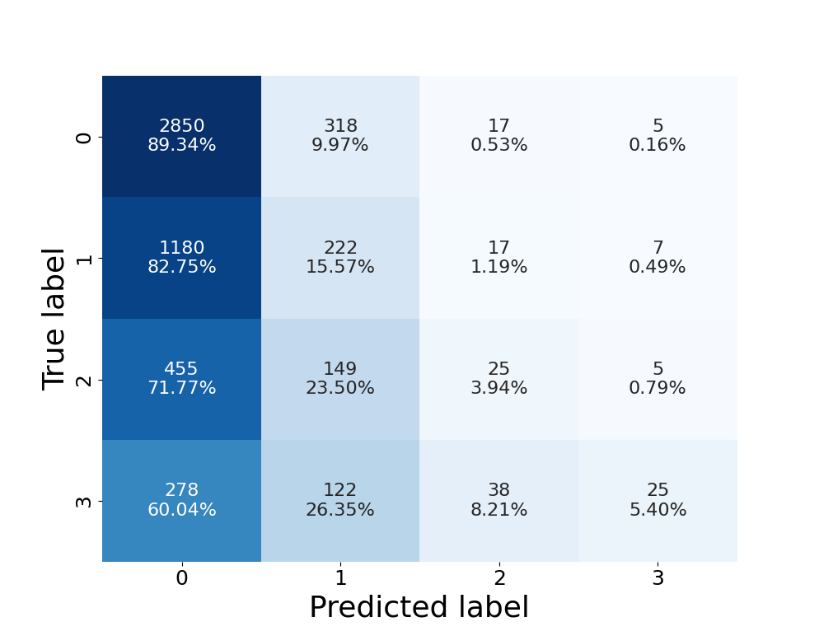


**A4. Classification matrices for GCS and ISS in all TBI patients (LR-ElasticNet)**
