# Supplementary material for: Utilizing ultra-early continuous physiologic data to develop automated measures of clinical severity in a traumatic brain injury population
Source: Sci Rep. 2024 Mar 31;14:7618. doi: 10.1038/s41598-024-57538-5 (PMC10982286; doi:10.1038/s41598-024-57538-5)
Supplement: Supplementary file 2 — Supplementary Information 2. [file 41598_2024_57538_MOESM2_ESM.docx]

Supplemental Figures


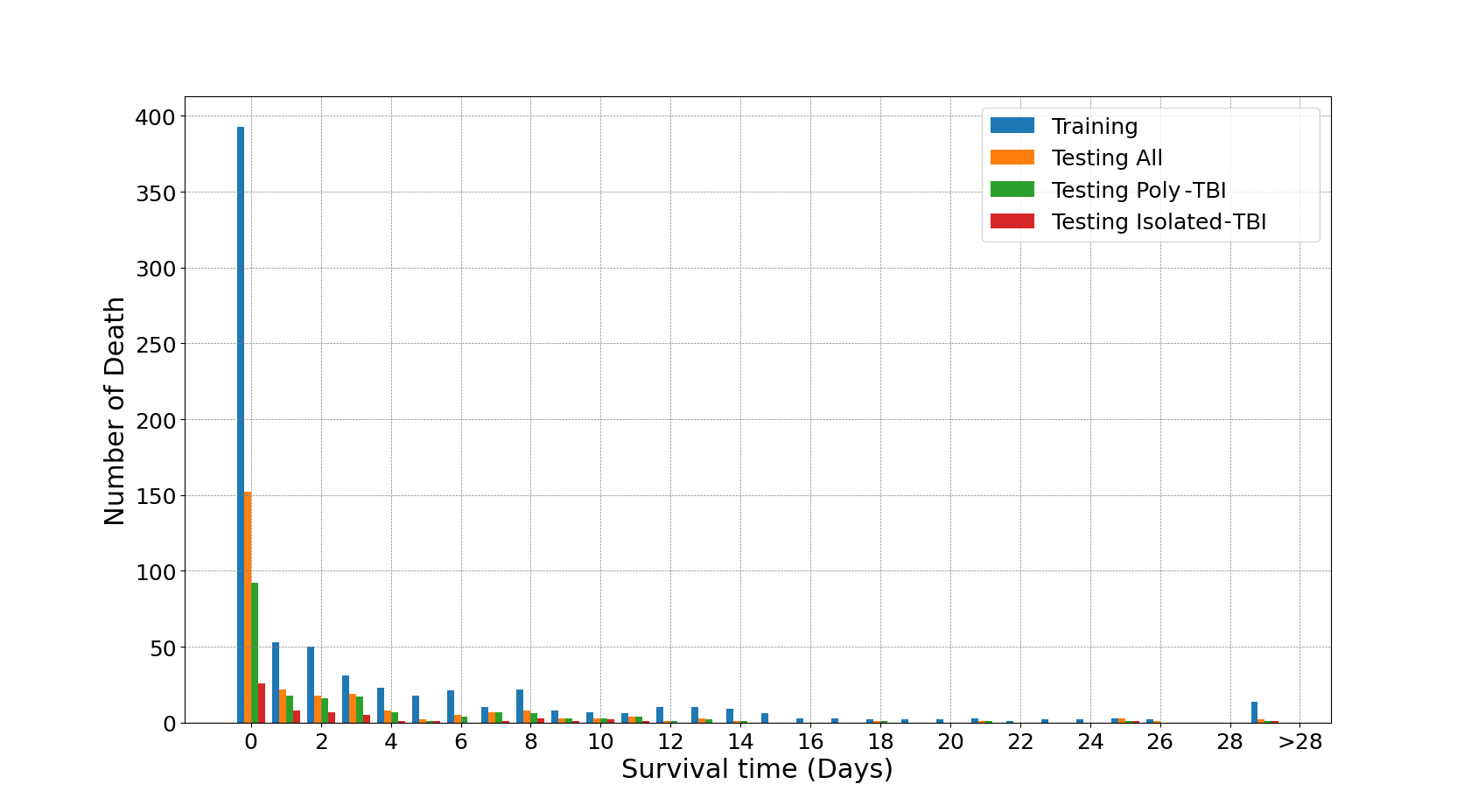


S1. Distribution of time of in-hospital mortality.


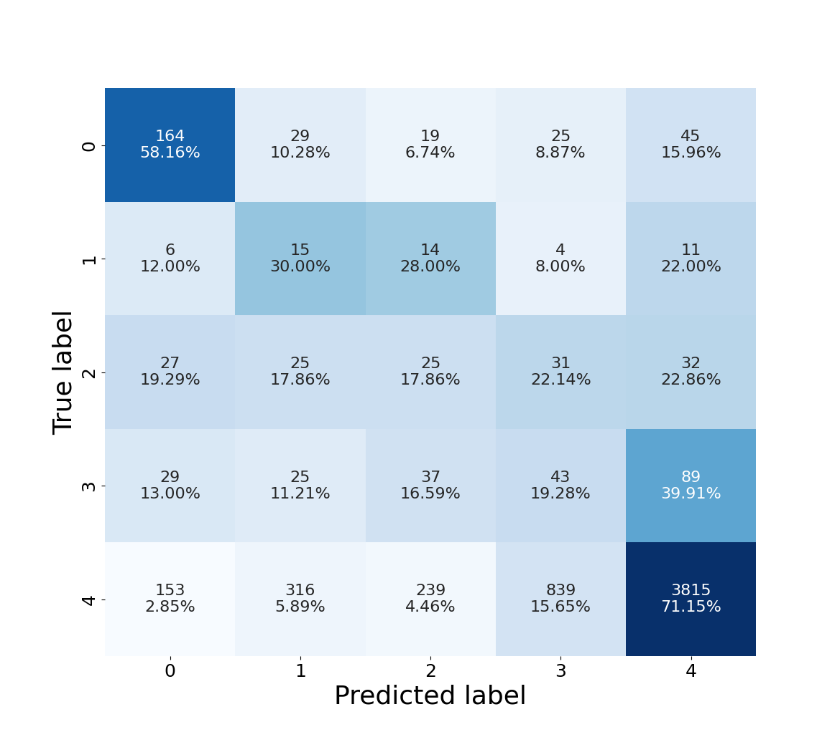

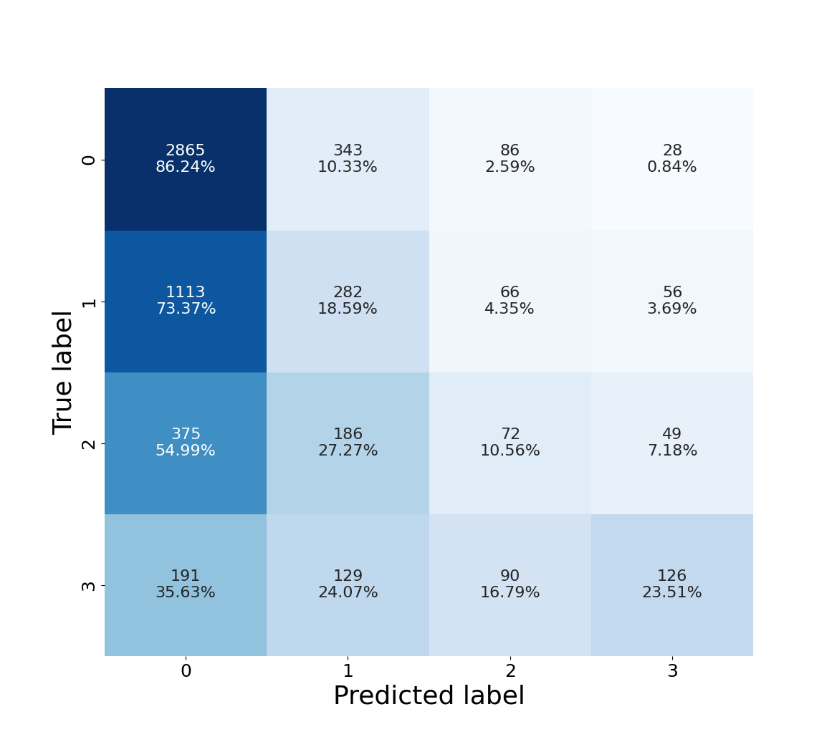


S2. Classification matrices for GCS and ISS in all TBI patients (XGBoost)


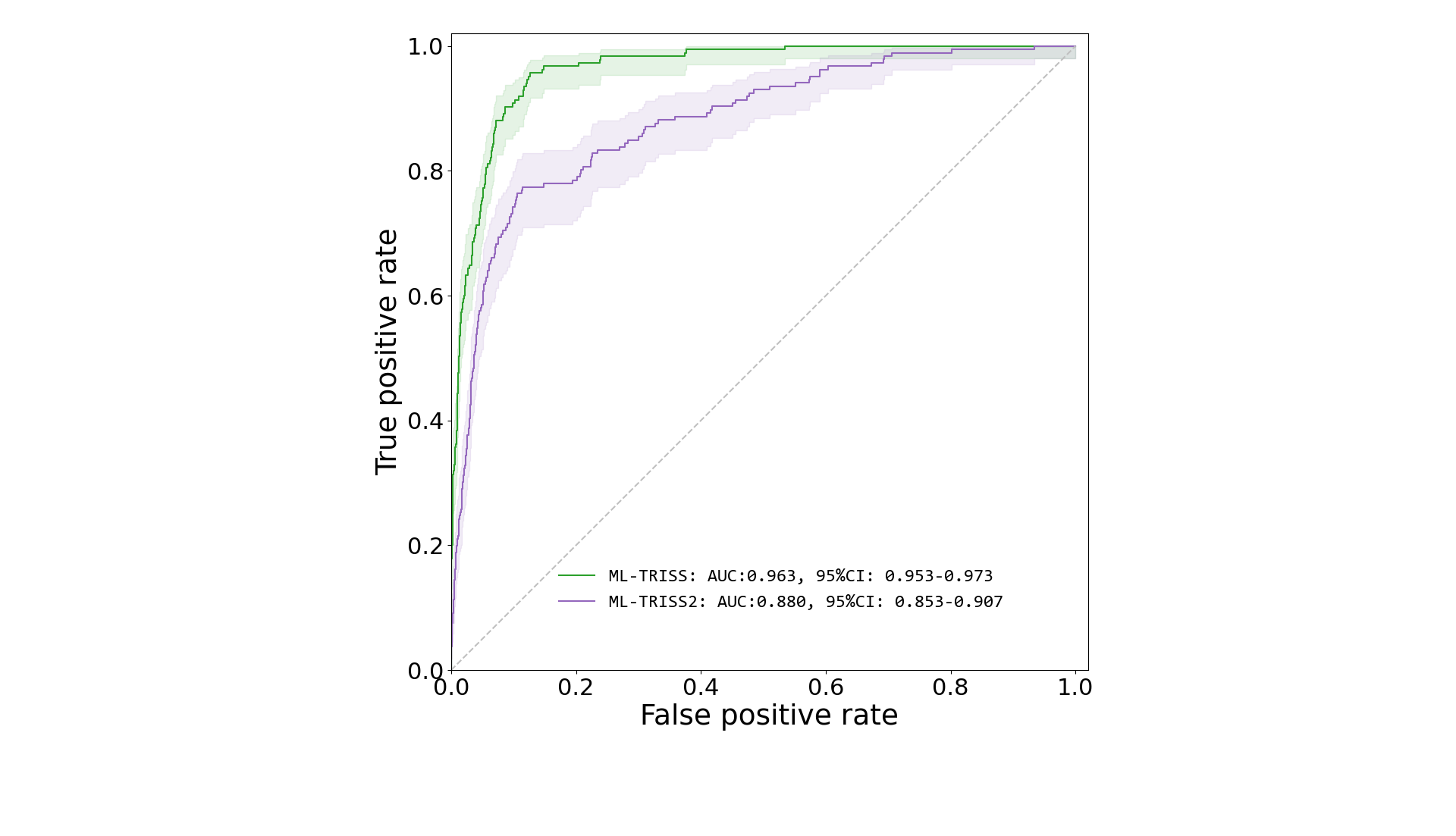


S3. Comparison of Mortality prediction using ML-TRISS (ML-ISS, real GCS) vs ML-TRISS-2 (ML-ISS, ML-GCS) in Poly-TBI

­


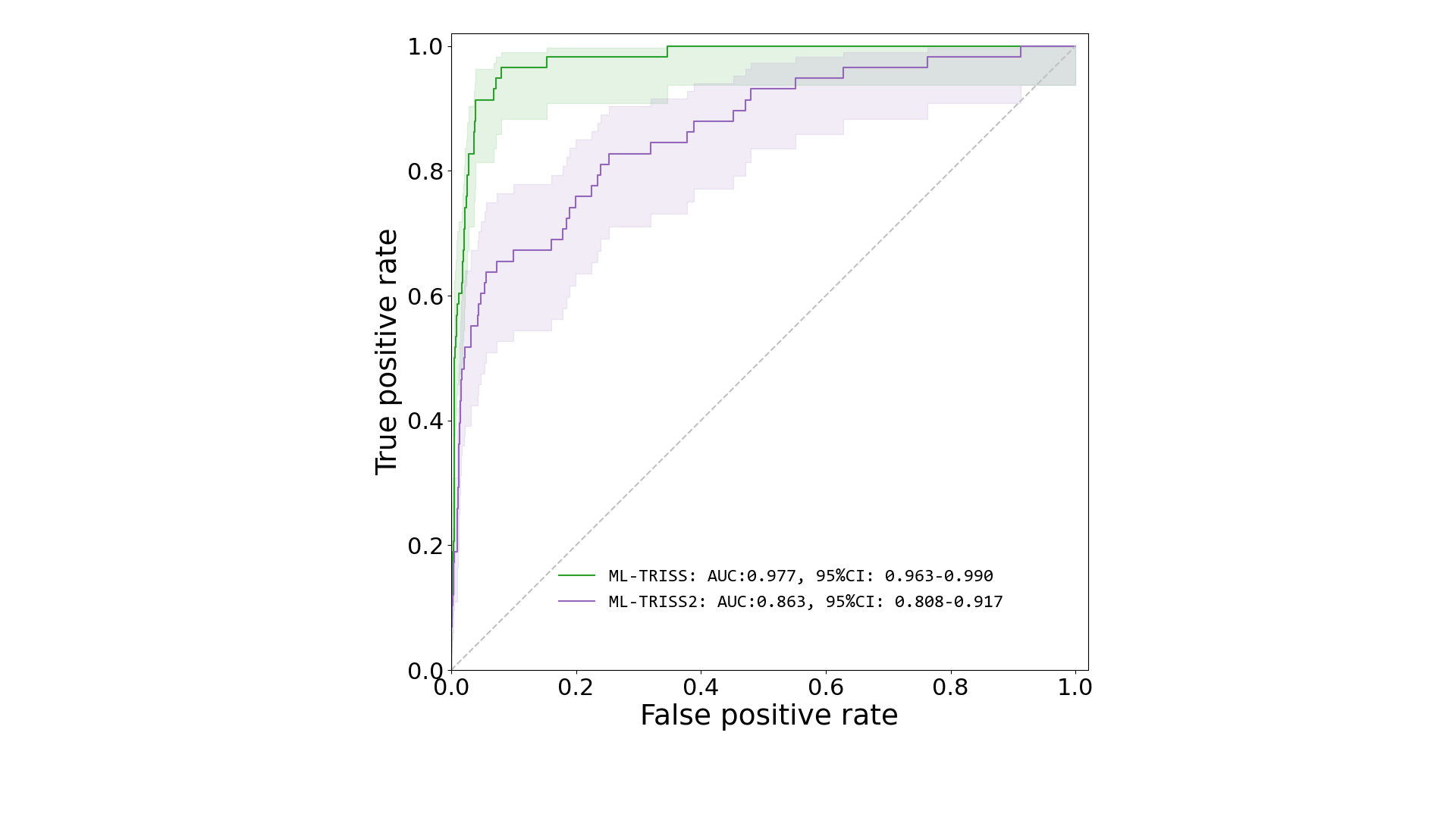


S4. Comparison of Mortality prediction using ML-TRISS (ML-ISS, real GCS) vs ML-TRISS-2 (ML-ISS, ML-GCS) in isolated TBI
